# Pre-radiotherapy neutrophil-to-lymphocyte ratio as an independent prognostic factor in patients with locally advanced hepatocellular carcinoma treated with radiotherapy

**DOI:** 10.18632/oncotarget.15209

**Published:** 2016-02-09

**Authors:** Seok Hyun Son, Eun Young Park, Hee Hyun Park, Chul Seung Kay, Hong Seok Jang

**Affiliations:** ^1^ Department of Radiation Oncology, Incheon St. Mary's Hospital, College of Medicine, The Catholic University of Korea, Seoul, Korea; ^2^ Department of Radiation Oncology, Seoul St. Mary's Hospital, College of Medicine, The Catholic University of Korea, Seoul, Korea

**Keywords:** neutrophil-to-lymphocyte ratio, radiotherapy, hepatocellular carcinoma

## Abstract

We aimed to investigate the pre-radiotherapy neutrophil-to-lymphocyte ratio (prNLR) as a prognostic factor in patients with locally advanced hepatocellular carcinoma (HCC) treated with radiotherapy (RT), and to determine the optimal cut-off value for prNLR. We retrospectively evaluated 56 patients with locally advanced HCC treated with RT (helical tomotherapy) between March 2006 and February 2012. The optimal cut-off value was determined by using a maximally selected log-rank test. Prognostic factors that influence the local progression-free survival (PFS) and overall survival (OS) were evaluated. A prNLR of 2.1 was determined to be the optimal cut-off value. In a comparison between the high-prNLR group and the low-prNLR group, there was a 13.1-month difference in the median OS (10.3 vs. 23.4 months, *p* = 0.003) and a 10.4-month difference in the median local PFS (7.1 vs. 17.5 months, *p* = 0.001). On multivariate analysis of prognostic factors for local PFS and OS, the prNLR was identified as an independent prognostic factor, and the hazard ratio was 4.2 and 2.5, respectively. We demonstrated that a low prNLR was significantly associated with better PFS and OS in patients with locally advanced HCC treated with RT, and the prNLR should be considered as an independent prognostic factor in these patients.

## INTRODUCTION

The standard treatments for unresectable locally advanced hepatocellular carcinoma (HCC) are transarterial chemoembolization (TACE) and sorafenib. TACE is currently recommended for large multinodular HCC [1, 2], whereas sorafenib is the suggested first-line treatment for HCC with vascular invasion or extrahepatic spread [3, 4]. Although radiotherapy (RT) has not been a main modality in the treatment of locally advanced HCC, several studies have suggested RT as an effective treatment option for patients with locally advanced HCC [5–7]. As RT has been more increasingly used with TACE or sorafenib, the knowledge of prognostic factors associated with RT has become important in the selection of optimal patients and in designing the RT strategy.

Recently, increasing evidences has shown that the presence of systemic inflammation correlates with poorer survival in some cancer patients [8–13]. Several studies have shown that the pretreatment neutrophil-to-lymphocyte ratio (NLR) was an independent prognostic factor in patients with HCC treated with surgical resection, transplantation, TACE, radiofrequency ablation (RFA), and sorafenib [10, 14–20]. The NLR is the ratio of the neutrophil count to the lymphocyte count, and is a useful index that reflects systemic inflammatory response in cancer patients [21, 22]. However, the range of previously reported cut-off values for NLR was from about 1.0 to 5.0, which is too wide for selecting an optimal and effective value in the real clinical setting. In addition, to our knowledge, there is no study on the prognostic role of NLR in patients with HCC treated with RT.

In this study, we aimed to investigate the pre-radiotherapy NLR (prNLR) as a prognostic factor in patients with locally advanced HCC treated with RT, and to determine the optimal cut-off value for the prNLR.

## RESULTS

### Optimal cut-off value for prNLR, and comparison between NLR groups stratified according to the cut-off value

The optimal cut-off value for predicting prognosis was calculated by using the maximally selected log-rank test, for which prNLR levels and local PFS were used as variables. According to the results of this statistical test, a NLR of 2.1 was determined to be a significant cut-off value of the prNLR. With this cut-off value, all 56 patients were divided in two groups: low-prNLR group (prNLR < 2.1) and high-prNLR group (prNLR ≥ 2.1). Sixteen patients (28.6%) were identified as the low-prNLR group, and the remaining 40 patients (71.4%) were identified as the high-prNLR group. The clinical characteristics were compared between the two prNLR groups, and described in Table 1. Age, sex, ECOG PS status, AJCC stage, presence or absence of hepatitis, liver cirrhosis, or PVTT, AFP levels, pretreatment CP class, and GTV were not different between the two NLR groups.

**Table 1:** Clinical characteristics associated with the level of prNLR

Variables	prNLR < 2.1	prNLR ≥ 2.1	*p* value
Sex			0.779
Male	11	29	
Female	5	14	
Age			0.538
Median	56	61	
Range	39–80	21–80	
ECOG PS			0.067
0	9	12	
1	7	28	
AJCC stage			0.257
II	4	4	
III	10	33	
IV	2	3	
Hepatitis			0.928
B	11	27	
Others	5	13	
Liver cirrhosis			0.350
No	6	10	
Yes	10	30	
PVTT			0.089
No	10	15	
Yes	6	25	
AFP			0.708
< 400	12	28	
≥ 400	4	12	
Pretreatment CP class			0.645
A	14	33	
B	2	7	
GTV			0.495
< 214 cm^3^	11	31	
≥ 214 cm^3^	5	9	

Abbreviations: prNLR, pre-radiotherapy neutrophil-to-lymphocyte ratio; ECOG PS, Eastern Cooperative Oncology Group performance status; AJCC, American Joint Committee on Cancer; PVTT, portal vein tumor thrombosis; AFP, alpha-fetoprotein; CP, Child-Pugh; GTV = gross tumor volume.

### Local PFS and OS

At the time of analysis, 13 patients (23.2%) developed actual local progression (1 patient: alive, 12 patients: deceased), 34 patients (60.7%) were deceased without evidence of local progression, and overall, 46 patients (82.1%) were deceased. The median follow-up duration was 13.2 months (range, 3.5–85.3 months). The median OS was 13.6 months, and the 1- and 2-year OS rates were 51.8% and 23.1%, respectively. The median local PFS was 10.5 months, and the 1- and 2-year local PFS rates were 42.4% and 12.6%, respectively.

### Clinical factors that influence the local PFS

In univariate analysis, female sex, presence of liver cirrhosis, AFP ≥ 400 IU/mL, pretreatment CP class B, and prNLR ≥ 2.1 were identified to be statistically significant unfavorable factors for local PFS (*p* = 0.028, 0.0015, 0.004, 0.035, and 0.001, respectively). Age, ECOG performance status, AJCC stage, presence or absence of hepatitis or PVTT, and GTV were not found to be statistically significant factors. In multivariate analysis, female sex, presence of liver cirrhosis, AFP ≥ 400 IU/mL, and prNLR ≥ 2.1 were identified to be statistically significant unfavorable factors for local PFS (*p* = 0.006, 0.020, 0.016, and < 0.001, respectively). The results of univariate and multivariate analyses are summarized in Table 2.

**Table 2:** Prognostic factors that influence the local progression-free survival

Variables	Univariate analysis		Multivariate analysis	
	HR (95% CI)	*p* value	HR (95% CI)	*p* value
Sex (Female)	2.045 (1.081–3.876)	0.028	2.566 (1.308–5.035)	0.006*
Age (≥ 60)	1.282 (0.956–1.721)	0.097		
ECOG PS (1)	1.297 (0.714–2.352)	0.393		
AJCC stage (II)	0.678 (0.196–2.343)	0.539		
AJCC stage (III)	1.009 (0.357–2.852)	0.986		
Hepatitis (B)	1.106 (0.549–1.879)	0.959		
Liver cirrhosis (presence)	2.427 (1.190–4.926)	0.015	2.456 (1.150–5.244)	0.020*
PVTT (presence)	1.742 (1.355–3.154)	0.067		
AFP (≥ 400)	2.570 (1.355–4.878)	0.004	2.281 (1.164–4.470)	0.016*
Pretreatment CP class (B)	2.487 (1.064–5.813)	0.035	2.340 (0.961–5.695)	0.061
GTV (≥ 214 cm^3^)	1.145 (0.592–2.217)	0.687		
Previous chemotherapy (presence)	0.621 (0.305–1.262)	0.188		
prNLR (≥ 2.1)	3.610 (1.680–7.751)	0.001	4.211 (1.930–9.188)	< 0.001*

Abbreviations: HR, hazard ratio; CI, confidence interval; ECOG PS, Eastern Cooperative Oncology Group performance status; AJCC, American Joint Committee on Cancer; PVTT, portal vein tumor thrombosis; AFP, alpha-fetoprotein; CP, Child-Pugh; GTV, gross tumor volume; prNLR, pre-radiotherapy neutrophil-to-lymphocyte ratio.

*Statistically significant.

### Clinical factors that influence the OS

In univariate analysis, age ≥ 60 years, presence of liver cirrhosis, AFP ≥ 400 IU/mL, and prNLR ≥ 2.1 were identified to be statistically significant unfavorable factors for OS (*p* = 0.035, 0.010, < 0.001, and 0.004, respectively). Sex, ECOG performance status, AJCC stage, presence or absence of hepatitis or PVTT, pretreatment CP class, and GTV were not found to be statistically significant factors. In multivariate analysis, presence of liver cirrhosis, AFP ≥ 400 IU/mL, and prNLR ≥ 2.1 were identified to be statistically significant unfavorable factors for OS (*p* = 0.048, < 0.001, and 0.023, respectively). The results of univariate and multivariate analyses are summarized in Table 3.

**Table 3:** Prognostic factors that influence the overall survival

Variables	Univariate analysis		Multivariate analysis	
	HR (95% CI)	*p* value	HR (95% CI)	*p* value
Sex (Female)	1.706 (0.904–3.215)	0.099		
Age (≥ 60)	1.919 (1.048–3.508)	0.035	1.417 (0.752–2.670)	0.281
ECOG PS (1)	1.161 (0.637–2.118)	0.624		
AJCC stage (II)	0.582 (0.169–2.009)	0.392		
AJCC stage (III)	0.720 (0.253–2.047)	0.537		
Hepatitis (B)	0.957 (0.516–1.776)	0.892		
Liver cirrhosis (presence)	2.557 (1.254–5.208)	0.010	2.065 (1.006–4.240)	0.048*
PVTT (presence)	1.438 (0.798–2.597)	0.226		
AFP (≥ 400)	3.597 (1.824–7.092)	< 0.001	3.605 (1.791–7.258)	< 0.001*
Pretreatment CP class (B)	1.538 (0.684–3.460)	0.297		
GTV (≥ 214 cm^3^)	0.834 (0.432–1.652)	0.605		
Previous chemotherapy (presence)	0.568 (0.279–1.155)	0.118		
prNLR (≥ 2.1)	2.941 (1.404–6.134)	0.004	2.474 (1.133–5.402)	0.023*

Abbreviations: HR, hazard ratio; CI, confidence interval; ECOG PS, Eastern Cooperative Oncology Group performance status; AJCC, American Joint Committee on Cancer; PVTT, portal vein tumor thrombosis; AFP, alpha-fetoprotein; CP, Child-Pugh; GTV, gross tumor volume; prNLR, pre-radiotherapy neutrophil-to-lymphocyte ratio.

*Statistically significant.

### Significance of prNLR in patients with locally advanced HCC treated with RT

**Figure 1:**
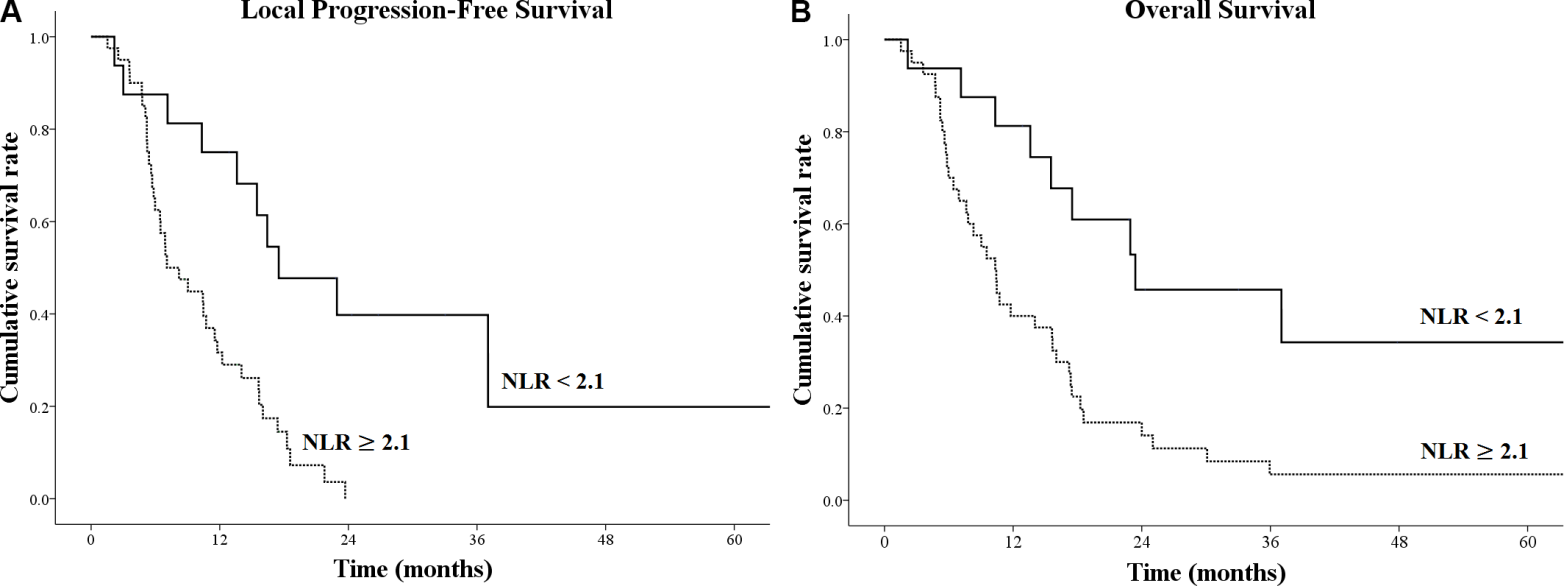
Kaplan-Meier survival curves for (**A**) local progression-free survival and (**B**) overall survival according to different levels of pre-radiotherapy neutrophil-to-lymphocyte ratio (prNLR).

In the low-prNLR group, the median OS was 23.4 months, and the 1- and 2-year OS rates were 81.3%, and 45.7%, respectively. The median local PFS was 17.5 months, and the 1- and 2-year local PFS rates were 75.0% and 39.8%, respectively (Figure 1). In the high-prNLR group, the median OS was 10.3 months, and the 1- and 2-year OS rates were 40.0% and 14.1%, respectively. The median local PFS was 7.1 months, and the 1- and 2-year local PFS rates were 29.0% and 0.0%, respectively (Figure 1). In a comparison between the high-prNLR and low-prNLR groups, there was a 13.1-month difference in the median OS (10.3 vs. 23.4 months, *p* = 0.003) and the relative risk of mortality was 2.5. There was 10.4-month difference in the median local PFS (7.1 vs. 17.5 months, *p* = 0.001), and the relative risk of disease progression was 4.2.

## DISCUSSION

NLR is a useful index that reflects systemic inflammatory response in some cancer patients [21, 22]. An increased neutrophil level was related to the systemic releases of chemokines and interleukins, which promote tumor growth and metastasis in HCC [23]. Lymphocytes are related to a T lymphocyte-mediated antitumor response [24], and a decreased lymphocyte number reflects a weaker lymphocyte-mediated immune response to the tumor [25]. Therefore, the NLR reflects the potential balance between neutrophil-associated pro-tumor inflammation and lymphocyte-dependent anti-tumor immune function; a high NLR is likely to reflect a more aggressive disease and poor prognosis [17, 26, 27].

Previous studies have investigated the value of NLR as a prognostic factor for HCC. The measurement of the NLR would be helpful in predicting the prognosis of malignant cancers such as gastric [12], lung [13], breast [8], pancreatic [11], and colorectal [9] cancers. In patients with HCC, a prognostic role of the NLR has been investigated after treatments including surgical resection [10, 17], transplantation [14], RFA [16], TACE [18–20], and sorafenib [15]. In addition, a recently reported meta-analysis demonstrated the role of the NLR [26]. This analysis revealed that low baseline NLR was significantly associated with better OS (hazard ratio [HR], 1.80; 95% confidence interval [CI], 1.59–2.04, *p* < 0.001) and recurrence-free or disease-free survival (HR, 1.80; 95% CI, 1.80–2.76, *p* < 0.001). Another finding was that low post-treatment NLR was significantly associated with better OS (HR, 1.90; 95% CI, 1.22–2.93, *p* < 0.001). In addition, a decreased NLR after treatment was significantly associated with better OS (HR, 2.23; 95% CI, 1.83–2.72, *p* < 0.001) and recurrence-free or disease-free survival of patients with HCC (HR, 2.23; 95% CI, 1.83–2.72, *p* < 0.001).

However, to our knowledge, there is no study on the prognostic role of the NLR in patients with HCC treated with RT. Therefore, in this study, we investigated the prognostic significance of the prNLR in patients with HCC who received helical tomotherapy with a hypofractionated scheme. This study showed the significant survival benefit in the low-prNLR group, which is consistent with several previous reports. In a comparison between high-prNLR and low-prNLR group, there was a 13.1-month difference in the median OS (10.3 vs. 23.4 months, *p* = 0.003) and a 10.4-month difference in the median local PFS (7.1 vs. 17.5 months, *p* = 0.001). On multivariate analysis of prognostic factors for local PFS and OS, the prNLR was identified as an independent prognostic factor, and the HR was 4.2 and 2.5, respectively.

To predict prognosis according to the NLR, determination of the cut-off value for the NLR is the most important point. In their study, Terashima et al. set the cut-off level of the NLR as the median value [19]. Another study determined the cut-off value by comparing the survival rates between NLR groups stratified according to several candidate values from 2.6 to 3.2 [28]. Several studies determined cut-off values by using receiver-operating characteristic (ROC) curves [17, 22]. In those studies, tumor response was selected as an endpoint to generate the ROC curve. However, those cut-off values were actually optimal for predicting tumor response, not for directly predicting survival. In this study, we chose a different statistical method to calculate the optimal cut-off value representing a direct association with local PFS. The cut-off value was calculated by using a maximally selected log-rank test, in which the presence or absence of local PFS, local PFS duration, and prNLR levels were used as variables [29]. Despite a different statistical method, the cut-off value of this study was similar to previously reported values. The range of reported cut-off values of the NLR was from about 1.0 to 5.0, which is too wide. According to the meta-analysis by Qi et al., the statistically significant cut-off value was 5.0 or within the range from about 2.0 to 3.0 [26]. Our cut-off value of 2.1 is within this range and was also statistically significant. Considering the statistical method for determining the cut-off value, our cut-off value is considered more optimal.

Li et al. reported that a low level of NLR was associated with lower AFP, ALP, and total bilirubin, as well as decreased incidences of ascites, portal vein thrombosis, and metastasis, all of which reflect better prognosis [22]. According to Okamura et al., lower AFP and smaller tumor size were associated with a low NLR [28]. Although the association between the NLR and other prognostic factors could be a valid explanation for the difference in prognosis, it was still controversial. In this study, there was no difference in other prognostic factors between the low-prNLR and high-prNLR groups. This absence of association with other prognostic factors could be a good explanation for why the prNLR should be considered an independent prognostic factor.

The results of this study should be carefully interpreted due to the retrospective nature of this study and the relatively small number of cases. Although our results were obtained using proper statistical analyses, they have not been sufficiently validated for generalization. Therefore, further large-scale validation studies are needed to confirm our results.

In conclusion, we demonstrated that a low prNLR was significantly associated with better PFS and OS in patients with locally advanced HCC treated with RT. The prNLR should be considered as another independent prognostic factor in case of RT.

## MATERIALS AND METHODS

### Patients

The inclusion criteria were as follows: 1) unresectable, locally advanced HCC; 2) age > 18 years; 3) a Child-Pugh (CP) score of 5, 6, or 7 within 1 month before RT; 4) an Eastern Cooperative Oncology Group (ECOG) performance status of 0 or 1; 5) an absence of distant metastases; 6) one or more laboratory tests before, during, and after RT; 6) one or more radiological evaluations before and after RT; and 7) a prescription dose of 40–50 Gy in 10 fractions.

A total of 56 patients were found to be eligible for this study. From March 2006 to February 2012, all patients received RT by using the TomoTherapy Hi-Art system (TomoTherapy Inc., Madison, WI, USA) at Incheon St. Mary's Hospital and Seoul St. Mary's Hospital. The patients’ data were retrospectively reviewed following institutional review board (IRB) approval (IRB of Incheon St. Mary's Hospital, reference no. OC16RISI0144).

Age, sex, ECOG performance status, American Joint Committee on Cancer (AJCC) stage (seventh edition), pretreatment CP class, absence or presence of hepatitis, liver cirrhosis, or portal vein tumor thrombosis (PVTT), alpha-fetoprotein (AFP) level, and prNLR were evaluated. Before RT, TACE was implemented in 49 patients (median number of procedures, 2; range, 1–11), percutaneous ethanol injection in 4 patients (median number of procedures, 2; range, 1–3), RFA in 7 patients (median number of procedures, 2; range, 1–3), and systemic chemotherapy in 12 patients. The patients’ characteristics are described in Table 4.

**Table 4:** Patients’ characteristics

Variables	*n*	%
Sex		
Male	40	71.4
Female	16	28.6
Age (years)		
Median	69	
Range	21–80	
ECOG PS		
0	21	37.5
1	35	62.5
Hepatitis		
None	6	10.7
HBV	38	67.9
HCV	5	8.9
Alcoholic	7	12.5
Liver cirrhosis		
No	16	28.6
Yes	40	71.4
PVTT		
No	25	44.6
Yes	31	55.4
AFP (IU/mL)		
< 400	40	71.4
≥ 400	16	28.6
CP class before radiotherapy		
A	47	83.9
B	9	16.1
AJCC stage		
II	8	14.3
III	43	76.8
IVA	5	8.9
Previous treatment		
None	6	12.5
TACE	49	87.5
RFA	7	12.5
PEI	4	7.1
Chemotherapy	12	21.4

Abbreviations: ECOG PS, Eastern Cooperative Oncology Group performance status; HBV, hepatitis B virus; HCV, hepatitis C virus; PVTT, portal vein tumor thrombosis; AFP, alpha-fetoprotein; CP class, Child-Pugh class; AJCC, American Joint Committee on Cancer; TACE, transarterial chemoembolization; RFA, radiofrequency ablation; PEI, percutaneous ethanol injection.

### Radiotherapy

For the simulations, the patients were immobilized by using the BodyFix system (Medical Intelligence GmbH, Schwabmunchen, Germany), in which the abdomen was compressed under low pressure with foil. Thereafter, contrast-enhanced computed tomography (CT) scans were performed with a 2.5-mm slice thickness on either a SOMATOM (Siemens, Berlin, Germany) or a LightSpeed RT16 (GE, Waukesha, WI, USA) CT scanner.

The gross tumor volume (GTV) was defined as the tumor volume that was enhanced in the arterial phase of the CT scan and diluted in the delayed phase. The planning target volume (PTV) was generated by the addition of 5–15 mm to the GTV according to the degree of respiratory movement. The organs at risk (OARs), such as the liver, stomach, duodenum, small and large intestines, both kidneys, and spinal cord, were also contoured.

The GTV was 127.4 ± 158.5 cm^3^, and the PTV was 329.5 ± 271.5 cm^3^. The total dose delivered to the PTV was 40–50 Gy (median, 50 Gy) in 10 fractions. The dose was prescribed to 95% of the PTV. The normal tissue constraints were as follows: 1) total liver volume receiving at least 20 Gy, < 60%; 2) mean liver dose, < 22 Gy; 3) mean kidney dose, < 13 Gy; 4) D_2cc_ (the dose to 2 cc volume of OARs) of the spinal cord, < 33 Gy; and 4) D_2cc_ of the stomach, duodenum, and intestine, < 35 Gy. We intended to perform the treatment plans based on the normal tissue constraints; however, these constraints were not always satisfied to achieve adequate target volume coverage. Treatment planning was performed by using the built-in software of the TomoTherapy Planning Station. We evaluated the dose-volume histogram and dose distributions in a slice-by-slice manner. We approved the treatment plan if the tumor coverage was adequate and the doses to the surrounding normal tissues were within clinically acceptable levels. Megavoltage cone-beam CT was performed during each treatment session before actual beam delivery. The patients’ set-up was corrected by using automated image registration, and the anatomical accuracy was evaluated by a radiation oncologist.

### Evaluation and statistical analysis

The effects of clinical factors, including age, sex, ECOG performance status, AJCC stage, presence or absence of liver cirrhosis, hepatitis, or PVTT, AFP level, pretreatment CP class, GTV, and prNLR, on the local progression-free survival (PFS) and overall survival (OS) were analyzed.

The OS duration was calculated from the date of RT to the date of death or the last follow-up date. The local PFS duration was calculated from the date of RT to the date of local disease progression, the last follow-up date, or the date of death. The local progression included the treated lesion only. The maximally selected log-rank test was used to determine the optimal cut-off value of prNLR [29]. The parameters used in this analysis were 1) the presence or absence of local PFS, 2) local PFS duration, and 3) prNLR levels. The cumulative survival was calculated using the Kaplan-Meier method. Univariate and multivariate analyses were performed by using Cox proportional hazards models (Probability for stepwise: Entry-0.05, Removal-0.10, Maximum iterations-20, Method: Enter). Significant variables found in univariate analysis were included in multivariate analysis. The association between the clinical characteristics and the prNLR group was analyzed by using the chi-square test and independent *t*-test. Statistical analysis was performed with R version 3.1.2 (R Development Core Team, Vienna, Austria), and *p* values < 0.05 were considered statistically significant.
